# Epidemiology of Subacute Sclerosing Panencephalitis (SSPE) in Germany from 2003 to 2009: A Risk Estimation

**DOI:** 10.1371/journal.pone.0068909

**Published:** 2013-07-09

**Authors:** Katharina Schönberger, Maria-Sabine Ludwig, Manfred Wildner, Benedikt Weissbrich

**Affiliations:** 1 Department of Public Health, Bavarian Health and Food Safety Authority, Oberschleissheim, Germany; 2 Department of Public Health, Bavarian Health and Food Safety Authority, Erlangen, Germany; 3 Institute of Virology and Immunobiology, Julius-Maximilian University of Würzburg, Würzburg, Germany; Institut Pasteur, France

## Abstract

Subacute sclerosing panencephalitis (SSPE) is a fatal long-term complication of measles infection. We performed an estimation of the total number of SSPE cases in Germany for the period 2003 to 2009 and calculated the risk of SSPE after an acute measles infection. SSPE cases were collected from the Surveillance Unit for Rare Paediatric Diseases in Germany and the Institute of Virology and Immunobiology at the University of Würzburg. The total number of SSPE cases was estimated by capture-recapture analysis. For the period 2003 to 2009, 31 children with SSPE who were treated at German hospitals were identified. The capture-recapture estimate was 39 cases (95% confidence interval: 29.2–48.0). The risk of developing SSPE for children contracting measles infection below 5 years of age was calculated as 1∶1700 to 1∶3300. This risk is in the same order of magnitude as the risk of a fatal acute measles infection.

## Introduction

Subacute sclerosing panencephalitis (SSPE) is a rare progressive, invariably fatal long-term complication of measles infection. The latency period between acute measles and first symptoms of SSPE is usually 4 to 10 years but ranges from 1 month to 27 years [1]. The clinical course of SSPE varies considerably in symptoms, duration and intensity. Typically, four disease stages are observed beginning with changes in personality and behaviour as well as failure in school. The second stage is characterized by massive, repetitive, and frequent myoclonic jerks, seizures and dementia. During the third stage, rigidity, extrapyramidal symptoms, and progressive unresponsiveness develop. The last stage is characterized by coma, a vegetative state, autonomic failure or akinetic mutism. The survival period after onset of symptoms is typically between one to three years [2], [3].

SSPE is caused by the intracerebral spread of measles virus leading to a destruction of neurons. In all cases where brain tissue has been examined by molecular methods, wild type measles virus strains have been identified, never vaccine strains [1], [4]. However, the exact pathogenesis of SSPE is still unclear. There is no evidence, that SSPE is associated with certain measles virus genotypes or strains. Immaturity of the immune system and of the central nervous system is thought to play a role when SSPE develops after an early acute measles infection [5]–[8].

Calculation of the risk of developing SSPE after measles infection is complex, because information on both measles and SSPE incidences is required. The calculation is additionally complicated by the long and variable latency period of SSPE. In the older literature, the risk of SSPE was reported to be about 1∶100000 cases of measles [9]. Later in a study from the UK, the risk of SSPE was estimated as 1 in 90000 measles cases in children of 5 years and older and as 1 of 5500 following measles infection in the first year of life [10], [11]. A more recent study from the USA reported a SSPE incidence of 6.5 to 11 cases per 100000 acute measles infections translating into a SSPE risk of 1 in 9100 to 15400 cases of measles [4]. A distinction of age groups was not made in this study.

The SSPE incidence may differ geographically and may change over time. Because there are only few current studies addressing the risk of SSPE, further studies are indicated in order to enlarge the epidemiological knowledge of this disastrous disease. For Germany, there is no notification or central documentation of SSPE cases. Therefore, information on the absolute number of SSPE cases and on the SSPE incidence in Germany is lacking. The aim of this study was to combine information on SSPE cases in Germany from different sources for the period 2003 to 2009, to estimate the total number of SSPE cases in children younger than 16 years of age in Germany and to calculate the risk of developing SSPE after an acute measles infection.

## Methods

### Ethics Statement

This study was approved by the ethics committee of the medical faculty at the University of Würzburg. Data collection and analysis within the Surveillance Unit for Rare Paediatric Diseases in Germany (ESPED) was approved by the ethics committee of the Bavarian Medical association. Informed consent by the patients or their guardians was not required by both ethics committees, because the data were analyzed anonymously.

### Data Sources of SSPE Cases

Information on SSPE cases for the period from 2003 to 2009 was obtained from two sources. The first dataset consisted of SSPE cases diagnosed at the Institute of Virology and Immunobiology at the Julius-Maximilian University Würzburg (ViroWue). Because of SSPE research interests, this institute has been involved in the diagnosis of SSPE for about 30 years. It is the appointed consultant laboratory for viral diseases of the central nervous system in Germany. From 1987 to 2010, samples of about 150 SSPE cases have been received from German hospitals for primary or confirmatory SSPE diagnosis.

The second data source was based on ESPED, a voluntary reporting system, which actively collects anonymous monthly reports from all German paediatric hospitals and departments on selected rare paediatric diseases. ESPED comprises a reminder and dunning system in order to collect missing reports. The return rate of monthly reports has been ≥95% since 1999 [12]. The spectrum of rare diseases, which are surveyed, has been changed on a regular basis. From 2003 to 2009, ESPED collected data about hospitalized children younger than 16 years of age with measles and its complications including SSPE. The Bavarian Health and Food Safety Authority (LGL) initiated and financed the survey. It was responsible for compilation of reported cases and for obtaining further information thereon. For that purpose, the LGL contacted the reporting paediatricians, who were asked to return an anonymous structured questionnaire about the case with questions on age, symptoms, diagnosis, complications, treatment and vaccination status. Additionally, an anonymized medical discharge letter was requested. The return rate of the questionnaires by the responsible physicians was 93.8% for this study.

### Data Sources of Measles Cases

Numbers of acute measles infections in Germany were obtained from two different official data sources. According to the German Infection Protection Act (IfSG), acute measles has been a notifiable disease since 2001. Data are collected by the health authorities of the federal German states and are reported to the Robert Koch-Institut (RKI), a central institution of the Federal Ministry of Health, which is responsible for disease control and prevention. Notification according to IfSG includes information about the hospitalization status of patients with acute measles. Notification data are publicly accessible (http://www3.rki.de). For the purpose of this study, numbers of notified acute measles infections for the period of 2001 to 2009 grouped by year, age, and hospitalization status were directly obtained from the RKI. These data allowed calculating a ratio between all measles cases and hospitalized measles cases (see below).

Before 2001, acute measles infection had not been a notifiable disease. Therefore, other indicators were sought in order to estimate the yearly numbers of measles infections. Since 1993, cumulated diagnosis data of hospitalized patients in Germany have been collected by the Federal Statistical Office as part of the German hospital statistics. These data have included information on hospitalized measles cases. All measles related hospitalizations covered by codes 055 of the International Classification of Diseases (ICD)-9 and codes B05 of ICD-10 were selected. For the year 1993, only incomplete data were available. For the years 1994 to 1999, hospitalization data on patients with measles, sorted by ICD and age groups, were directly obtained from the Federal Statistical Office. For the years 2000 to 2009, data on hospitalized measles cases were obtained from a publicly accessible online-database (http://www.gbe-bund.de). Data from the German hospital statistics were only available in clustered age groups (1994–1999: age groups 0 to <5 and ≥5 to <15 years of age; 2000–2009: age groups 0 to <5, ≥5 to <10, and ≥10 to <15 years of age).

### Case Definition, Data Merging and Validation

Laboratory diagnosis of SSPE at ViroWue was based on established criteria including oligoclonal bands, intrathecal measles virus-specific IgG synthesis and excessively high absolutes titres of measles virus IgG in both serum and CSF. In each case, the laboratory diagnosis was complemented by clinical findings that were consistent with SSPE. Reports of SSPE cases by the ESPED surveillance system were checked for plausibility by contacting the reporting paediatrician. Fulfilment of diagnostic criteria for SSPE was confirmed based on discharge reports. Discharge reports were not available for two cases captured only by ESPED (number 19 and 30 in table 1).

**Table 1:** Characteristics of subacute sclerosing panencephalitis (SSPE) cases in Germany from 2003 to 2009.

No	Data source	yearreported	sex	nationality	year ofbirth	year ofprimary SSPEdiagnosis	age at SSPEdiagnosis	year ofmeaslesinfection	age atmeaslesinfection	country ofmeaslesinfection	latency perioduntil SSPEdiagnosis (years)	number ofvaccinedoses	age at first/secondvaccination
1	ViroWue, ESPED^a^	2003	male	Kosovan	1996	2003	7	n. i.	n. i.	n. i.	n. i.	2	5/7
2	ViroWue, ESPED	2003	male	Turkish	1997	2003	6	1998	1	born in Germany^b^	5	n. i.	n. i.
3	ViroWue, ESPED	2003	male	Turkish	1995	2003	8	n. i.	n. i.	born in Germany^b^	n. i.	2	1/4
4	ViroWue	2003	male	Iraqi	1994	2003	9	1996	2	n. i.	7	2	3/7
5	ViroWue	2003	female	n. i.	1995	2003	8	n. i.	n. i.	n. i.	n. i.	n. i.	n. i.
6	ESPED	2003	male	Croatian	1996	2003	7	1997	1	n. i.	6	n. i.	n. i.
7	ESPED	2004	male	German	1998	2004	6	1999	1	Germany^b^	5	1	2
8	ViroWue	2004	n. i.	n. i.	1996	2004	8	n. i.	n. i.	n. i.	n. i.	2	3/6
9	ViroWue a, ESPED	2004	male	German	1997	2002	5	1998	1	Germany^b^	4	n. i.	n. i.
10	ViroWue a, ESPED	2004	female	n. i.	1992	2002	10	1994	2	n. i.	8	n. i.	n. i.
11	ViroWue, ESPED	2005	male	German	1995	2005	10	1996	1	Germany^b^	9	2	7/8
12	ViroWue, ESPED	2005	male	Angolan	1992	2005	13	1993	1	n. i.	12	1	12
13	ViroWue, ESPED	2005	female	German	1995	2005	10	1996	1	Germany^b^	9	1	1
14	ViroWue	2005	female	Turkish	1995	2005	10	1996	1	n. i.	10	n. i.	n. i.
15	ViroWue	2005	female	German	1995	2005	10	1995	0	Germany^b^	10	2	n. i.
16	ViroWue	2005	n. i.	n. i.	1996	2005	9	n. i.	n. i.	n. i.	n. i.	n. i.	n. i.
17	ViroWue	2005	male	n. i.	1997	2005	8	n. i.	n. i.	n. i.	n. i.	n. i.	n. i.
18	ESPED	2005	male	German	1994	2005	11	1995	1	Germany^b^	10	n. i.	n. i.
19	ESPED	2005	male	Kosovan	1994	2005	11	n. i.	n. i.	n. i.	n. i.	1	n. i.
20	ESPED	2005	male	Turkish	1995	2005	10	1996	1	n. i.	9	2	1/4
21	ViroWue, ESPED	2005	male	Turkish	2004	2005	1	n. i.	n. i.	n. i.	n. i.	1	1
22	ViroWue	2006	male	n. i.	2001	2006	5	2001	1	Germany^c^	4	2	1/n. i.
23	ViroWue	2006	male	n. i.	1993	2006	13	n. i.	n. i.	n. i.	n. i.	n. i.	n. i.
24	ViroWue	2006	male	German	1997	2006	9	1998	1	Germany^b^	8	n. i.	n. i.
25	ViroWue	2006	male	Tamil	1998	2006	8	1999	<1	Germany^c^	7	n. i.	n. i.
26	ViroWue, ESPED	2007	female	German/Romanian	1998	2007	9	1999	1	Germany^b^	8	n. i.	n. i.
27	ESPED	2007	female	Turkish	1999	2007	8	2000	1	n. i.	7	3	n. i.
28	ViroWue, ESPED	2008	female	German	2005	2008	3	2006	1	Germany^b^	2	1	2
29	ViroWue	2008	male	German	1996	2008	12	1997	1	Germany^b^	11	1	1
30	ESPED	2008	male	German	1998	2008	10	1999	1	Germany^b^	9	2	7/10
31	ViroWue	2009	male	n. i.	1997	2009	12	n. i.	n. i.	n. i.	n. i.	n. i.	n. i.

abbreviations: n. i. – no information, ESPED - Surveillance Unit for Rare Paediatric Diseases in Germany, ViroWue - Institute of Virology and Immunobiology at the University of Würzburg,

a reported to ESPED in 2004, samples tested at ViroWue in 2002.

b country of infection assumed to be Germany because of German nationality and/or birth in Germany.

c country of infection Germany based on anamnestic information.

Because the ESPED data were limited to children younger than 16 years of age during the period 2003 to 2009, we restricted our study of SSPE cases to this period and age group. Cases from both SSPE data sources were matched and merged based on information contained in both data sets (month and year of birth, second letter of pre- and surname, time and city of hospitalization). Matching of cases was performed independently by two investigators of the study (KS and BW) yielding identical results. For all uniquely identified cases, information on acute measles infection was used for further analysis if available. Children with SSPE from abroad who were only hospitalized in Germany for diagnostic purposes were excluded from the analysis. However, children residing in Germany were included independent of their ethnic background.

### Calculation of Total Number of SSPE Cases by Capture-recapture Method

To estimate the total number of SSPE cases in Germany during the period 2003 to 2009, we used the capture-recapture method. Both SSPE data sources collected the data simultaneously and independently in accordance with the requirements for this method. The capture-recapture estimator and its confidence interval were calculated according to LaPorte et al. [13].

### Calculation of the SSPE Risk after Known Measles Infection

For the age group below 5 years of age, the SSPE cases in the numerator were taken from the years 2003 to 2009 (study period). The corresponding measles cases were taken from the years 1994 to 2001 as “at-risk”-population (years of known measles infections).

## Results

### SSPE Case Identification and Capture-recapture Calculation

After merging the SSPE cases from ViroWue and ESPED, 38 unique SSPE cases who were diagnosed at ViroWue and/or reported to ESPED at some point during 2003 to 2009 were identified. Of these, four cases were excluded because they had no residence in Germany and another three cases because they were older than 16 years of age at the time of SSPE diagnosis. The remaining 31 cases fulfilled the case definition and were included in further analysis (table 1). Of these 31 cases, 11 (31%) were captured by both data sources, 13 (42%) were captured exclusively by ViroWue, and seven (23%) exclusively by the ESPED surveillance system. Based on information available to ViroWue and ESPED, all children had their primary SSPE diagnoses at the time they were captured, except for cases number 9 and 10 (table 1), who were both captured by ESPED in 2004 but were primarily diagnosed with SSPE in 2002, when ESPED was not yet collecting cases with measles complications. By capture-recapture analysis, the estimated total number of SSPE cases in Germany during the period 2003 to 2009 was 39 (95% confidence interval [CI]: 29.2–48.0; table 2) in children younger than 16 years of age.

**Table 2:** Capture-recapture estimation of subacute sclerosing panencephalitis (SSPE) cases in Germany from 2003 to 2009.

	ViroWue	ESPED	Captured by both data sources	Sum of captured cases	Capture-recapture estimation (95% CI)
All children with SSPE of this study	24^a^	18	11^a^	31	38.6 (29.2–48.0)
Children with SSPE, who possibly contracted measles infection in Germany 1994–2001^b^	13	12	6	19	25.0 (16.3–33.7)
Children with SSPE, who probably contracted measles infection in Germany 1994–2001b, c	10	8	5	13	15.5 (10.7–20.3)

abbreviations: ESPED - Surveillance Unit for Rare Paediatric Diseases in Germany, ViroWue - Institute of Virology and Immunobiology at the University of Würzburg, CI – confidence interval.

a Cases 9 and 10 of table 1 (captured by ESPED in 2004, samples tested by ViroWue in 2002) were included.

b Children with missing information on year of measles infection were excluded.

c German children and/or children born in Germany. Children with missing information on nationality and/or country of birth or infection were excluded.

### SSPE Case Descriptions

**Figure 1:**
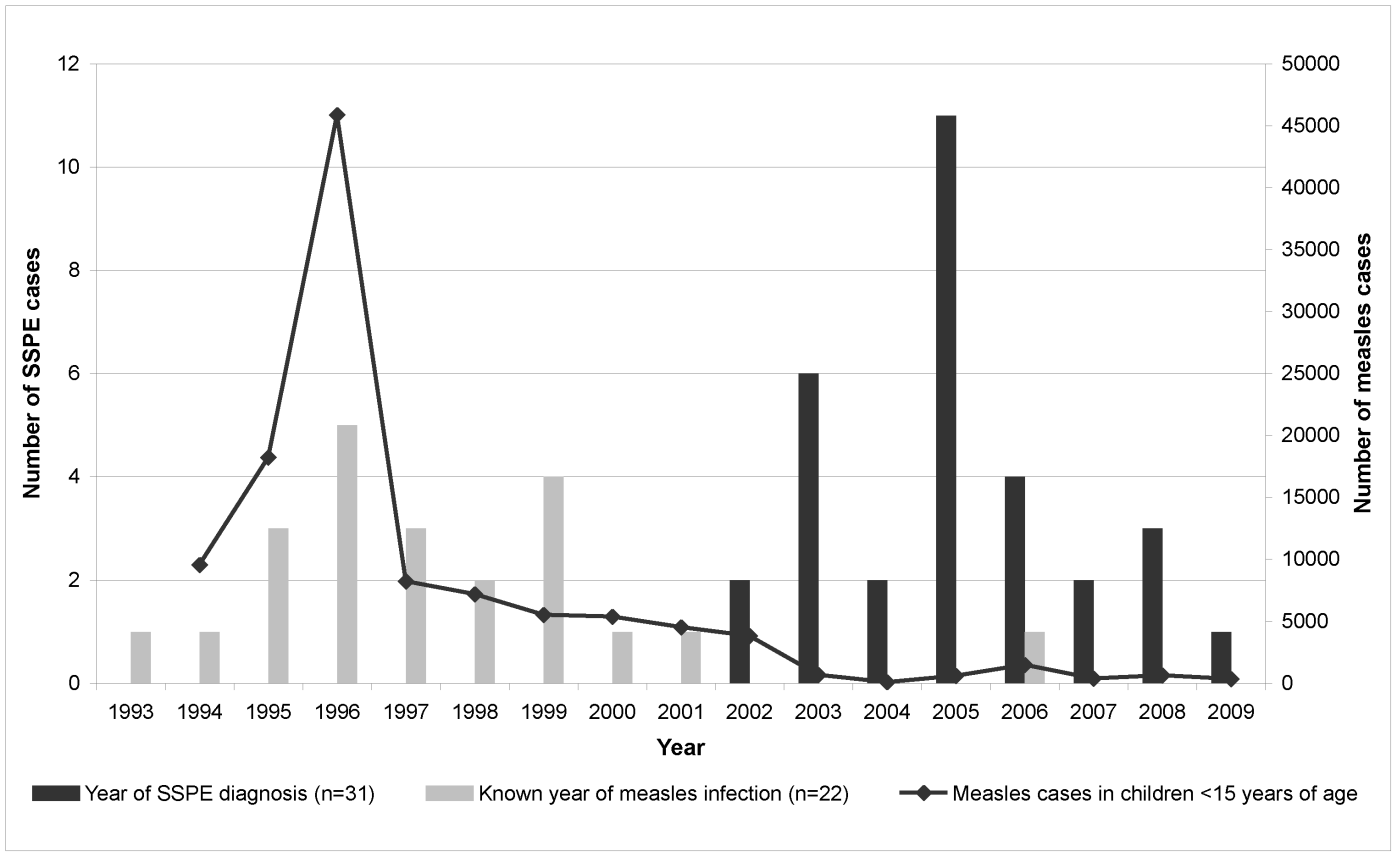
Year of primary diagnosis of subacute sclerosing panencephalitis (SSPE) and year of measles infection. Data are shown for SSPE cases identified in Germany from 2003 to 2009. Two SSPE cases reported to the German Surveillance Unit for Rare Paediatric Diseases (ESPED) in 2004 were primarily diagnosed in 2002. Numbers of measles cases are extrapolated based on German hospital statistics for the period 1994–2000. For the period 2001–2009, measles cases reported by the German Infection Protection Act (IfSG) are displayed.

Twenty-one of the 31 children with SSPE were male and 8 were female (male-to-female ratio 2.6∶1). The gender of two patients was unknown. Information about nationality was available for 23 of the 31 cases; 11 children were German, 6 Turkish, 2 Kosovarian, and 4 had other non-German nationalities (table 1). The median and mean age at SSPE diagnosis was 9 years (range 1 to 13 years). Of the 21 cases with a known history of measles infection, the median and mean age at time of measles infection was 1 year and all children were younger than 3 years of age. The median latency period between measles infection and SSPE diagnosis was 8 years (mean 7.6 years; range 2 to 12) (table 1). A yearly distribution of the acute measles infections of these 21 children is shown in figure 1. Except for one case of measles infection in 2006, all other infections occurred during the period of 1993 to 2001. Information about the vaccination status and/or the age at the time of measles vaccination was available for 17 children. Insofar as it was known, vaccination took place later than infection with the measles virus.

### Calculation of the Risk of SSPE after Acute Measles Infections

For the children with SSPE and known history of measles in the period 1993 to 2001, calculation of the risk to develop SSPE was attempted. To this end, knowledge of the total number of measles cases as denominator is necessary. As mentioned above, acute measles has been a notifiable disease in Germany only since 2001 as regulated by IfSG. Since 1993, however, the Federal Statistical Office has collected data on the number of hospitalized measles cases as part of the German hospital statistics. Because these data were incomplete for the starting year 1993 of hospital statistics, this year was excluded from further calculations.

Based on the assumption, that the ratio between hospitalized cases and all measles cases was nearly constant during the period of 1994 to 2009, we determined this ratio on the basis of all IfSG notifications from 2001 to 2009 (“pooled IfSG hospitalization rate”). Using this ratio, we extrapolated the number of all measles cases for the period from 1994 to 2001 from German hospital statistics data. This extrapolation was performed for the age group of children below 5 years of age, because all children with SSPE and known history of measles infection were below 5 years of age at the time of acute measles infection. The pooled IfSG hospitalization rate was 0.092 for this age group (table 3). Using this rate, we extrapolated the number of measles cases per year from the numbers of hospitalized cases reported by the German hospital statistics. In total, approximately 43000 measles cases were assumed to have occurred in Germany from 1994 to 2001 in children younger than 5 years of age (table 3).

**Table 3:** Extrapolation of the total number of measles cases from 1994 to 2009 in Germany for children <5 years of age ^a.^

Year	Reported measlescases in children <5years (IfSG data)	Hospitalized measlescases in children<5 years (IfSG data)	Hospitalizationrate (IfSG data)	Hospitalized measlescases in children<5 years (GHS data)	Measles cases in children <5years extrapolated from GHSdata using the IfSGhospitalization rate
1994	no data available	no data available		383	*4163*
1995	no data available	no data available		691	*7511*
1996	no data available	no data available		1628	*17696*
1997	no data available	no data available		284	*3087*
1998	no data available	no data available		276	*3000*
1999	no data available	no data available		213	*2315*
2000	no data available	no data available		175	*1902*
2001	1839	161	0.088	265	*2880*
2002	1542	94	0.061	153	*1663*
2003	285	13	0.046	34	*370*
2004	60	9	0.150	22	*239*
2005	207	20	0.097	33	*359*
2006	536	86	0.160	103	*1120*
2007	126	12	0.095	29	*315*
2008	196	22	0.112	28	*304*
2009	165	37	0.224	36	*391*
**Total 1994–2000**				**3650**	***39674***
**Total 2001–2009**	**4956**	**454**	**0.092**	**703**	***7641***
**Total 1994–2001** b				**3915**	***42554***

abbreviations: IfSG - German Infection Protection Act, GHS – German hospital statistics.

a Numbers in columns 2–5 are reported data; numbers in column 6 (in italics) represent extrapolations from GHS data using the pooled hospitalization rate derived form IfSG data for the years 2001 to 2009 (454/4956 = 0.092).

b Time period used for SSPE risk estimation.

The validity of this approach was assessed for the period 2001 to 2009, during which both data sources (notification by IfSG and German hospital statistics) were available (table 3). The total number of IfSG measles notifications was 4956. The total number of measles cases extrapolated from German hospital statistics using the pooled IfSG hospitalization rate was 7641. This approach was further validated in the age group≥5 and <15 years (table 4). Overall, 7525 children with measles infection were reported by IfSG and 344 of these were hospitalized, yielding a pooled IfSG hospitalization rate of 0.046. Using this rate, the number of measles infections extrapolated from German hospital statistics was 11112. Thus, measles case extrapolations from German hospital statistics and the number of IfSG measles notifications were in fairly good agreement in both age groups with German hospital statistics derived numbers being about 1.5fold higher than IfSG notifications.

**Table 4:** Extrapolation of the total number of measles cases from 1994 to 2009 in Germany for children ≥5 and <15 years of age^a^.

Year	Reported measles cases in children≥5 and <15 years(IfSG data)	Hospitalized measlescases in children≥5 and <15 years (IfSGdata)	Hospitalizationrate (IfSG data)	Hospitalized measlescases in children ≥5 and<15 years (GHS data)	Measles cases in children ≥5and <15 years extrapolatedfrom GHS data using the IfSGhospitalization rate
1994	no data available	no data available		247	*5403*
1995	no data available	no data available		490	*10719*
1996	no data available	no data available		1288	*28175*
1997	no data available	no data available		234	*5119*
1998	no data available	no data available		191	*4178*
1999	no data available	no data available		147	*3216*
2000	no data available	no data available		158	*3456*
2001	2692	109	0.040	177	*3872*
2002	2271	93	0.041	141	*3084*
2003	369	12	0.033	17	*372*
2004	25	2	0.080	11	*241*
2005	386	16	0.041	21	*459*
2006	961	70	0.073	86	*1881*
2007	240	9	0.038	16	*350*
2008	427	18	0.042	18	*394*
2009	154	15	0.097	21	*459*
**Total 1994–2000**				**2755**	***60266***
**Total 2001–2009**	**7525**	**344**	**0.046**	**508**	***11112***

abbreviations: IfSG - German Infection Protection Act, GHS – German hospital statistics.

a Numbers in columns 2–5 are reported data; numbers in column 6 (in italics) represent extrapolations from GHS data using the pooled hospitalization rate derived form IfSG data for the years 2001 to 2009 (344/7525 = 0.046).

Using the number of measles cases extrapolated from German hospital statistics for the period 1994 to 2001 as denominator, the risk of developing SSPE was calculated for children with measles infection below 5 years of age. With respect to the numerator, i. e. the number of SSPE cases, we made various assumptions (table 5). When all captured children with SSPE were considered, for whom the year of measles infection was known and the country of infection was possibly Germany (n = 19), the incidence of SSPE was 1 of 2240 measles infections. Using the capture-recapture estimate as denominator (n = 25), the incidence was 1 of 1702 measles infections. Given the fact, that precise information on the country of measles infection was not available for all SSPE cases, we performed similar calculations with restriction to the children of German nationality and/or children born or anamnestically infected in Germany (n = 13). The ensuing SSPE incidence was 1 of 3273 measles cases, which increased to 1 of 2660 measles cases when the capture-recapture estimate of SSPE cases (n = 16) was used.

**Table 5:** Estimation of the risk of subacute sclerosing panencephalitis (SSPE) in Germany after acute measles during the period 1994 to 2001^a^.

Assumption on measles infection^b^	Source of SSPE cases^b^	Number of SSPE cases (2003–2009)	Age at acute measles	Extrapolated total number of measles cases (1994–2001)	SSPE risk	SSPE risk per 100000 measles infections
possibly in Germany	captured cases	19	<5 years	42554	1 of 2240	44.6
	capture-recapture estimation	25	<5 years	42554	1 of 1702	58.8
probably in Germany	captured cases	13	<5 years	42554	1 of 3273	30.6
	capture-recapture estimation	16	<5 years	42554	1 of 2660	37.6

a The estimation was based on children with SSPE diagnosis during 2003 to 2009, who had a history of acute measles infection during 1994 to 2001.

b Details are explained in Table 2.

## Discussion

We estimated the risk of developing SSPE after acute measles infection below 5 years of age to be in the range of 1 in 1700 to 1 in 3300 cases of measles infection. Because SSPE is not a notifiable disease in Germany nor were acute measles infections notifiable before 2001, several assumptions were made for the risk calculations. Concerning the number of SSPE cases, we focused on the years 2003 to 2009 because two independent data sources were available for this period. One data source was ESPED, a voluntary reporting system, which has actively collected monthly reports from all German paediatric hospitals and departments on a predefined spectrum of rare paediatric diseases. Measles and its complications – including SSPE – had been part of the ESPED survey from 2003 to 2009. The second data source was provided by ViroWue, which is the consultant laboratory for viral diseases of the central nervous system in Germany and receives samples of SSPE patients for confirmatory diagnosis on a voluntary basis. Because ViroWue is only one of many laboratories in Germany offering diagnostic tests for SSPE, it is unclear in how far these cases are representative for the whole of Germany. Availability of these two data sources for the years 2003 to 2009 provided the unique opportunity of a capture-recapture-analysis of SSPE cases in Germany for this period. Both data sources combined, 31 SSPE cases had been observed. By capture-recapture estimation, the total number of SSPE cases for the period 2003 to 2009 was 39 (95% CI: 29.2–48.0). Thus, this analysis provided evidence that both data sources had collected about 84% but at least more than half of all SSPE cases from German hospitals in children younger than 16 years of age. Consequently, the SSPE cases captured by ESPED and ViroWue were assumed to be representative for the German situation and could therefore be used for calculations of the risk of SSPE after measles infection.

Capture-recapture estimations are useful to compensate for the insufficiency of surveillance systems. They are based on five central assumptions: (1) cases have to be clearly identifiable as cases, (2) double notifications of identified cases have to be clearly detectable, (3) cases have to be descended from a closed population, (4) homogeneity in capture of cases, and (5) independence of both data sets [14]. These five prerequisites were considered to be reasonably fulfilled, because (1) diagnostic criteria for SSPE are clearly defined, (2) unequivocal case matching was possible based on the available information from both data sources, (3) analysis was restricted to German hospitals and patients living in Germany, (4) severity of SSPE symptoms with progression suggests that disease diagnosis will be attempted in all cases, and (5) independence of data sets was assumed because both collection systems were fundamentally different with ViroWue being part of the diagnostic and confirmatory process and ESPED being a reporting system for already diagnosed cases. However, positive dependence of both capture systems is not unlikely, because diagnosis of SSPE is a prerequisite for the disease to be reported by ESPED as demonstrated, e.g., by cases 9 and 10. Because positive dependence will result in underestimation of the true population size, the effect on SSPE risk calculation will equally be underestimation. Nevertheless, even if the assumption of independence was violated, capture-recapture analysis would still be useful by providing a lower limit of the SSPE cases [15].

The available information about the SSPE cases was heterogeneous. Yet, the overall demographic characteristics of our cohort with a male preponderance and median age of 9 years were in good agreement with data from the literature [review in 1,2,16]. Information about a history of acute measles infection was available for 21 children with SSPE. These children provided the basis of calculations of the SSPE risk. Their median age at the time of measles infection (1 year; range 0–2 years) as well as the latency period until diagnosis of SSPE (median 8 years; range 2–12 years) were again in accordance with the results from other studies [review in 1,2,16]. Precise information on the country where acute measles infection took place was not available for most of the children with SSPE. This aspect is discussed below.

Concerning the denominator of the SSPE risk calculations, i.e. the number of cases with acute measles infections, we were faced with the problem that most of the measles infections in the cohort of our SSPE patients took place before the start of mandatory measles notification in Germany in 2001. Therefore, extrapolation of the total number of measles cases in the 1990s was attempted on the basis of hospitalized acute measles cases recorded through the German hospital statistics system starting in 1993. Comparison of data derived from IfSG and the German hospital statistics for the period 2001 to 2009 provided evidence that this approach was valid.

Yearly hospitalization rates derived from IfSG data of the years 2001 to 2009 varied by a factor of about 5 for the age group<5 years and by a factor of about 3 for the age group≥5 and <15 years (column 4 in tables 3 and 4). Therefore, we used the pooled hospitalization rate, which is mainly influenced by the years with large numbers of measles cases and is more robust against variation seen in years with small numbers of measles cases. This has two additional effects. Firstly, the data from the German hospital statistics suggest that during the IfSG reporting period, the measles epidemiology of the years 2001 and 2002 is most similar to the period 1994 to 2000 when IfSG reporting was not yet instituted. Because 2001 and 2002 were the years with the largest numbers of measles cases reported by IfSG, they mainly influence the pooled hospitalization rate. This indicates, that the pooled IfSG hospitalization rate is representative for the period 1994 to 2000. Secondly, the hospitalization rates for the years 2001 and 2002 and as a result the pooled hospitalization rate are comparatively low (column 4 in Tables 3 and 4). When extrapolating the number of measles cases from the German hospital statistics, the effect of using a low hospitalisation rate is a tendency to overestimate measles cases.

Based on the combined cohorts of SSPE patients and the estimated yearly numbers of acute measles cases, the risk of developing SSPE was calculated. For the children with known history of acute measles, almost all infections took place in the period of 1994 to 2001 and all children were younger than 3 years of age at the time of acute measles. Several of these children had a migration background and information on where acute measles infection took place was not available for all cases. The conservatively calculated SSPE risk taking into account only children infected probably in Germany was 1 in 3300 cases of acute measles in children below 5 years of age. The risk increased to 1 in 2240 when children possibly infected with measles virus in Germany were added.

Calculation of the SSPE risk is limited by uncertainties with respect to the number of acute measles cases and the number of SSPE cases. Underestimation of the number of acute measles cases may lead to overestimation of the risk of SSPE. In how far this may have occurred is unclear. In a study of the 2006 measles outbreak in Germany, IfSG notification data were compared with billing data received by statutory health insurance carriers from physicians [17]. Based on this comparison, under-reporting by IfSG in the age group of <5 years of age was estimated to be about fourfold while there appeared to be considerably less underreporting in all other age groups. This striking difference between age groups remained unexplained. Furthermore, it is unclear whether the billing data were more reliable than IfSG notification data, because in the former there may be a tendency to report suspected measles cases as confirmed measles cases. At any rate, the use of measles case numbers extrapolated from German hospital statistics in our study appeared to decrease the effect of under-reporting, which may be inherent to IfSG data, because numbers derived from German hospital statistics were about 1.5fold higher than IfSG notifications.

Underestimation of the number of SSPE cases may lead to underestimation of the actual risk of SSPE. Several factors suggest, that underestimation of SSPE cases is a relevant issue. Firstly, some of the patients without known history of measles infection may have had acute measles in the period from 1994 to 2001. Secondly, further SSPE cases resulting from measles infections in the period 1994 to 2001 may have had their primary diagnosis before or after the observation period 2003 to 2009 of this study. Thirdly, only SSPE cases in children younger than 16 years of age were captured for this study because of restrictions of the data collection system (ESPED inclusion criteria). And finally, capture-recapture analysis suggested that the true number of SSPE diagnoses from 2003 to 2009 was probably higher than observed. In addition, the age-specific risk of SSPE is likely to be higher for children below 3 years of age, because all children with known history of measles were 2 years or younger at the time. However, extrapolation of the total number of measles cases was only possible in 5-year-age groups due to limitations imposed by the German hospital statistics with respect to age-specific data. Within the IfSG notifications, the proportion of children below 3 years of age among the age group of children below 5 years of age is 59.6% for all cases of measles and 81.5% for hospitalized cases of measles. From these data, it can be estimated that the SSPE risk for children below 3 years of age was about 1.7fold higher than the risk for the larger group of children below 5 years of age.

Unfortunately, the risk of SSPE for children above 5 years of age at the time of measles infection could not be calculated for two reasons. On the one hand, data about measles virus infections for the period 1994 to 2001 were available only as clustered numbers for the age group 6 to 15 years of age. On the other hand, SSPE cases were collected only for children younger than 16 years of age by ESPED. Because of the long latency period of SSPE it is evident, that SSPE cases resulting from measles infections in children at the age of 6 to 15 years would, at best, be only partially detected by ESPED.

Nevertheless, the age distribution at the time of acute measles in our study suggests that the risk of developing SSPE is particularly high after measles infection in the first two to three years of life and decreases thereafter. This is in line with previous observations and highlights the importance of vaccination against measles at the earliest age recommended by national guidelines [1], [10], [11], [18]. Measles vaccination provides protection against SSPE as long as wild-type measles infection does not occur before vaccination [1]. However, due to the presence of maternal antibodies, the recommended age for the first measles vaccination is usually around the end of the first year of life. Prevention of SSPE by protection from wild-type measles infection in children below 1 year of age can only be achieved by herd immunity ensuing from sufficient vaccination coverage population wide. This is an important aspect in the context of the elimination of measles, because the risk of developing SSPE may be in the order of 1∶1000 measles cases or even higher after measles infection in the first year of life.

In conclusion, this study provides data on the SSPE epidemiology in Germany for the period 2003 to 2009. Our data suggest that the risk of developing SSPE after acute measles infection below 5 years of age is in the range of 1∶1700 to 1∶3300. This considerable risk for a disastrous yet preventable disease should be incorporated in every risk-benefit-education of measles vaccination and may constitute an important component of communication about the rationale for measles elimination.
